# The Science and Art of Surgery

**Published:** 1878-08

**Authors:** 


### BIBLIOGRAPHICAL.

THE SCIENCE AND ART OF SURGERY, being a Treatise on Surgical Injuries, Diseases and Operations. By John Eric Erichsen, F.R.S., F.R.C.S., Surgeon Extraordinary to her Majesty the Queen, etc., etc. Revised by the author from the seventh and enlarged edition. Illustrated with eight hundred and sixty-two engravings on wood. In two vols. octavo, aggregating about two thousand pages. Philadelphia: Henry C. Lea. 1878.

This valuable text-book of Surgery having been enlarged and revised by the author, may be said to be indispensable to the completeness of a medical library.

